# Electroacupuncture-Like Stimulation at the Baihui (GV20) and Dazhui (GV14) Acupoints Protects Rats against Subacute-Phase Cerebral Ischemia-Reperfusion Injuries by Reducing S100B-Mediated Neurotoxicity

**DOI:** 10.1371/journal.pone.0091426

**Published:** 2014-03-13

**Authors:** Chin-Yi Cheng, Jaung-Geng Lin, Nou-Ying Tang, Shung-Te Kao, Ching-Liang Hsieh

**Affiliations:** 1 School of Chinese Medicine, College of Chinese Medicine, China Medical University, Taichung, Taiwan; 2 Department of Chinese Medicine, Hui-Sheng Hospital, Taichung, Taiwan; 3 Acupuncture Research Center, China Medical University, Taichung, Taiwan; 4 Department of Chinese Medicine, China Medical University Hospital, Taichung, Taiwan; 5 Graduate Institute of Integrated Medicine, College of Chinese Medicine, China Medical University, Taichung, Taiwan; National University of Singapore, Singapore

## Abstract

**Objectives:**

The purpose of this study was to evaluate the effects of electroacupuncture-like stimulation at the Baihui (GV20) and Dazhui (GV14) acupoints (EA at acupoints) during the subacute phase of cerebral ischemia-reperfusion (I/R) injury and to establish the neuroprotective mechanisms involved in the modulation of the S100B-mediated signaling pathway.

**Methods:**

The experimental rats were subjected to middle cerebral artery occlusion (MCAo) for 15 min followed by 1 d or 7 d of reperfusion. EA at acupoints was applied 1 d postreperfusion then once daily for 6 consecutive days.

**Results:**

We observed that 15 min of MCAo caused delayed infarct expansion 7 d after reperfusion. EA at acupoints significantly reduced the cerebral infarct and neurological deficit scores. EA at acupoints also downregulated the expression of the glial fibrillary acidic protein (GFAP), S100B, nuclear factor-κB (NF-κB; p50), and tumor necrosis factor-α (TNF-α), and reduced the level of inducible nitric oxide synthase (iNOS) and apoptosis in the ischemic cortical penumbra 7 d after reperfusion. Western blot analysis showed that EA at acupoints significantly downregulated the cytosolic expression of phospho-p38 MAP kinase (p-p38 MAP kinase), tumor necrosis factor receptor type 1-associated death domain (TRADD), Fas-associated death domain (FADD), cleaved caspase-8, and cleaved caspase-3 in the ischemic cortical penumbra 7 d after reperfusion. EA at acupoints significantly reduced the numbers of GFAP/S100B and S100B/nitrotyrosine double-labeled cells.

**Conclusion:**

Our study results indicate that EA at acupoints initiated 1 d postreperfusion effectively downregulates astrocytic S100B expression to provide neuroprotection against delayed infarct expansion by modulating p38 MAP kinase-mediated NF-κB expression. These effects subsequently reduce oxidative/nitrative stress and inhibit the TNF-α/TRADD/FADD/cleaved caspase-8/cleaved caspase-3 apoptotic pathway in the ischemic cortical penumbra 7 d after reperfusion.

## Introduction

During the subacute phase of ischemic brain injury (1–7 d after the onset of ischemia), astrocytes become activated and accumulate in the periinfarct area, leading to reactive astrogliosis and glial scar formation, which exacerbate delayed infarct expansion and play a key pathological role in ischemic injury [1], [2]. The S100B protein, containing two helix-loop-helix calcium-binding structural motifs, exerts differing effects on neurons depending on its concentration: neurotrophic effects at nanomolar concentrations and neurotoxic effects at micromolar concentrations [3]. Increasing evidence has shown that an increase in the synthesis of S100B by activated astrocytes in the periinfarct area is positively associated with the severity of delayed infarct expansion and neurological deficits in models of middle cerebral artery occlusion (MCAo) [1], [4], whereas S100B inhibitors exert potential neuroprotective effects against cerebral ischemic injuries [5], [6]. Thus, the S100B is an effective biomarker of the severity of infarction and the extent of brain edema, as shown by previous clinical [7] and experimental stroke [8] studies. During the subacute phase of cerebral ischemia, astrocytes release S100B, which interacts with the receptor for advanced glycation end products (RAGE) to stimulate the microglial secretion of proinflammatory cytokines, such as interleukin-1β (IL-1β) and tumor necrosis factor-α (TNF-α), by activating nuclear factor-κB (NF-κB). The activated microglia then triggers astrocytic activation by releasing IL-1β and TNF-α, which promote S100B overexpression [1].

Previous studies have shown that extracellular S100B elicits complex neuron-glial interactions at high concentrations, and upregulates inducible nitric oxide synthase (iNOS) expression and nitric oxide (NO) production in a RAGE-dependent manner in glial cells to cause NO diffusion and neurotoxicity [1], [9], [10], [11]. The S100B also induces apoptotic cell death through a NO-dependent pathway in cultured astrocytes [11] and neurons [12] in vitro. A study by Yasuda et al. showed that NO can be highly toxic because of secondary reactions that generate peroxynitrite and hydroxyl free radicals, which contributed to the expansion of cerebral infarction in a model of transient MCAo [13].

Chinese physicians have used acupuncture for the treatment of stroke patients for several centuries [14], [15]. A randomized controlled trial by Sallstrom et al. has shown that acupuncture can provide significant therapeutic benefits to stroke patients including by improving daily life activities and motor function during the subacute stage [16]. According to traditional Chinese medicine theory, the Baihui (GV20) and Dazhui (GV14) acupoints are both on the “Du meridian”, which directly communicates with the brain, and are commonly used to treat stroke. Experimental studies in rats have shown that EA at the Baihui acupoint can reduce cerebral edema during the acute phase [17] and exert protective effects against caspase-3-dependent neuronal apoptosis during the subacute phase [18], of cerebral ischemia. Luo et al. further reported that EA at the Baihui and Dazhui acupoints exerts beneficial effects on neural regeneration and synaptic reconstruction through the modulation of reactive astrocytosis in the cortical penumbra in the subacute and chronic phases of the permanent MCAo model [19]. Therefore, the purpose of our study was to evaluate the effects of EA-like stimulation at the Baihui and Dazhui acupoints (EA at acupoints) after 15 min of cerebral ischemia followed by 7 d of reperfusion, and to elucidate the regulatory mechanisms involved in the S100B-mediated signaling pathway in the periinfarct area following ischemia-reperfusion (I/R) injury.

## Materials and Methods

### Experimental Animals

Male Sprague Dawley (SD) rats weighing 300–350 g (approximately 8–9 weeks of age) were used for the experiments. The humidity levels were between 55±5%, and the rats were maintained on a 12 h light-dark cycle at 22±2°C. All experimental procedures were performed in accordance with the guidelines approved by the China Medical University Institutional Animal Care and Use Committee (Permit Number: 100-215-c).

### Model of MCAo

The model of MCAo was established in the SD rats using an intraluminal suture method as described previously [20]. Briefly, the rats were anesthetized with chloral hydrate (400 mg/kg, intraperitoneally), then placed in a supine position, and the right common carotid artery (CCA) and the internal carotid artery (ICA) were exposed through a neck midline incision, before ligation of the pterygopalatine artery close to its branch. A 3–0 nylon monofilament suture, blunted at the tip by a flame and coated with poly-L-lysine (Sigma, USA), was inserted into the right external carotid artery (ECA) through the CCA into the ICA for 20–25 mm to prevent blood flow into the middle cerebral artery (MCA). The suture was removed slowly to reestablish the blood flow 15 min after the MCAo. The rectal temperature of the rats was maintained at 37±0.5°C throughout the surgical procedure using an electric heating pad.

### Electrode Implantation

After the completion of the MCAo operation, the head of each rat was fixed to a stereotactic frame and its scalp or costal skin was incised. The electrode consisted of 0.5-mm stainless steel wires used for acupoint (or nonacupoint) stimulation. The electrode was implanted in the Baihui (midpoint of the parietal bone, 4 mm depth, forward insertion) and Dazhui (below the spinous process of the seventh cervical vertebra, 5 mm depth, vertical insertion) acupoints, or in the bilateral costal regions (nonacupoints). The rat was then returned to its cage.

### Assessment of Neurological Function

The neurological function of each rat was assessed after 1 d and 7 d of reperfusion. The motor, sensory, balance, and reflex functions were evaluated using the modified neurological severity score as described previously [21]. The neurological function of each rat was graded using a numeric scale from 0 to 18 (reference score, 0; maximal deficit score, 18). Except in the sham-operation group, the rats with neurological deficit scores ≥7 after 1 d of reperfusion were included in subsequent analyses, whereas rats with neurological deficit scores <7 were excluded.

## Experiment A

### Grouping

The rats were randomly divided into Sham-1 d, Model-1 d, Sham-7 d, Model-7 d, EA-7 d, and Non-acup-7 d groups (n = 5 or 6). The rats in the EA-7 d group were subjected to 15 min of MCAo. After 1 d of reperfusion, the rats received EA at acupoints once daily for 6 consecutive days. They were sacrificed 7 d after reperfusion. The rats in the Non-acup-7 d group were subjected to the same procedures as the rats in the EA-7 d group but received EA at nonacupoints. The rats in the Model-1 d group were subjected to 15 min of MCAo, and then sacrificed after 1 d of reperfusion. The rats in the Model-7 d group were subjected to the same procedures as the rats in the EA-7 d group but did not receive EA. The rats in the Sham-1 d group were subjected to the same procedures as the rats in the Model-1 d group but the MCA origin was not occluded. The rats in the Sham-7 d group were subjected to the same procedures as the rats in the Model-7 d group but the MCA origin was not occluded.

### EA at Acupoints or Nonacupoints

An EA apparatus (Trio 300, ITO Co, Germany) was used to generate EA at acupoints or nonacupoints for 25 min once daily for 6 consecutive days. The stimulation parameters were a 5 Hz amplitude-modulated wave, 2.7–3.0 mA intensity, and a 150-µs pulse width. The rats were awake and moving freely in their cages during EA at acupoints or nonacupoints.

### Measurement of the Cerebral Infarct Area

Following their neurological examination 1 d or 7 d after reperfusion, the rats were sacrificed under deep anesthesia. The brains were removed immediately and cut into 2-mm sections using a brain matrix. The sections were then stained with 2% 2,3,5-triphenyltetrazolium chloride (TTC; Merck, Germany) for 15 min at 37°C. The brain tissue was differentiated according to staining: white for infarct areas and red for noninfarct areas. The cerebral infarct areas of the first 6 sections from the frontal lobe were measured using image analysis software (ImageJ, Java). The ratios of infarct areas to the total brain areas were calculated.

## Experiment B

The rats were randomly divided into 4 groups: EA-7 d, Non-acup-7 d, Model-7 d, and Sham-7 d groups. They were then subjected to the experimental procedures described in Experiment A.

### Immunohistochemical (IHC) Analysis

After 15 min of cerebral ischemia followed by 7 d of reperfusion, the rats were sacrificed under deep anesthesia (n = 5 or 6). They were then transcardially perfused with 200 ml of a 0.9% saline and 200 ml of a 4% paraformalaldehyde (PFA; pH 7.4). The rat brains were removed immediately and postfixed in a 4% PFA followed by 30% sucrose (weight/volume) for 3 d, after which they were cut into 15-µm sections using a cryostat. The brain sections were rinsed with a Dulbecco’s phosphate buffered saline (DPBS; Sigma-Aldrich) containing 0.01% Tween-20 and immersed in 3% hydrogen peroxide (H_2_O_2_)/methanol for 15 min for the inhibition of endogenous peroxidase activity. They were then incubated with a 10% normal animal serum (ScyTek, Logan, Utah, USA) for 20 min at room temperature (RT) prior to incubation in moist chambers with a mouse anti-glial fibrillary acidic protein (GFAP; 1∶200 dilution, IF03L Calbiochem), rabbit anti-S100B (1∶1000 dilution, NB110-57478 Novus Biologicals), rabbit anti-NF-κB (p50; 1∶100 dilution, sc-114 Santa Cruz), rabbit anti-TNF-α (1∶100 dilution, BMS175 Bender MedSystems), or mouse anti-iNOS (1∶200 dilution, N32020 Transduction Laboratories) antibody overnight at 4°C. Following incubation with the appropriate secondary antibody and avidin-biotin peroxidase complexes (ABC kit, ScyTek, Logan, Utah, USA), the sections were colored using a 3,3′-diaminobenzidine (DAB) kit (ScyTek, Logan, Utah, USA), and counterstained with hematoxylin. The stained sections were mounted in a mounting medium (Assistant-Histokitt, Germany) and the immunoreactive cells in the ischemic cortical penumbra were analyzed under a light microscope (Axioskop 40, Zeiss). The GFAP-, S100B-, NF-κB (p50)-, TNF-α-, and iNOS-stained adjacent serial sections from the Model-7 d group incubated without primary antibodies were used as negative controls.

### IHC Costaining

The brain sections were immersed in 3% H_2_O_2_/methanol for 15 min and then incubated with a diluted normal blocking serum (Vector Laboratories, CA, USA) for 25 min at RT. The sections were then incubated with a mouse anti-GFAP (1∶200 dilution, IF03L Calbiochem), or a mouse anti-nitrotyrosine (1∶100 dilution, MAB5404 Chemicon) antibody for 1.5 h at 37°C and washed with the DPBS. Following their incubation with the diluted biotinylated secondary antibody and an ABC-AP reagent (AK-5002, Vectastain), the sections were stained with an alkaline phosphatase substrate solution (SK-5300, Vector Blue). They were then incubated with a rabbit anti-S100B antibody (1∶1000 dilution, NB110-57478 Novus Biologicals) for 1.5 h at 37°C and washed with the DPBS. Following their incubation with the diluted biotinylated secondary antibody and an ABC-AP reagent (AK-5001, Vectastain), the sections were stained with an alkaline phosphatase substrate solution (SK-5100, Vector Red), dried, and mounted in the mounting medium. Finally, the immunoreactive cells in the ischemic cortical penumbra were analyzed under a light microscope.

### Terminal Deoxynucleotidyl Transferase-mediated dUTP-biotin Nick-end Labeling (TUNEL) Assay

TUNEL staining was performed according to the manufacturer’s instructions (QIA33, Calbiochem, USA). Briefly, the brain sections adjacent to those used in IHC analysis were incubated with 20 µg/ml proteinase K for 20 min at RT, rinsed with a Tris-buffered saline, and incubated with a 1× terminal deoxynucleotidyl transferase (TdT) equilibration buffer for 30 min at RT. The sections were then incubated with a TdT labeling reaction mixture for 1.5 h at 37°C. After the addition of the stop solution and the blocking buffer, the sections were incubated with a 1× conjugate solution for 30 min at RT, and the TUNEL-reactive cells were visualized using a DAB kit (Calbiochem). Finally, the immunoreactive cells in the ischemic cortical penumbra were evaluated under a light microscope.

### Immunofluorescent (IF) Costaining

The brain sections were incubated with a diluted normal blocking serum (Vector Laboratories, CA, USA) for 20 min at RT, and then incubated with a rabbit anti-RAGE (1∶200 dilution, ab3611 Abcam) or rabbit anti-NF-κB (p50; 1∶50 dilution, sc-114 Santa Cruz) antibody overnight at 4°C. After washing 3 times with the DPBS, the sections were incubated with a DyLight 488-conjugated AffiniPure goat anti-rabbit IgG antibody (green, 1∶400 dilution, Jackson ImmunoResearch) for 1 h at RT. The NF-κB (p50)-stained sections were then counterstained with 4′,6-diamidino-2-phenylindole (DAPI; Sigma-Aldrich, USA, nuclear staining) for 10 min at RT. The remaining RAGE-stained sections were incubated with a mouse anti-S100 (1∶200 dilution, ab4066 Abcam) antibody overnight at 4°C, and then incubated with a DyLight 594-conjugated AffiniPure goat anti-mouse IgG antibody (red, 1∶400 dilution, Jackson ImmunoResearch) for 1 h at RT. Finally, all sections were *mounted* in an aqueous *mounting medium (Aquatex, HC886685 Merck) and* viewed under a fluorescent microscope (CKX41, Olympus). Sections incubated without the RAGE and S100 primary antibodies provided the IF costaining negative controls.

### Western Blot Analysis

Seven days after reperfusion, the rats were anesthetized with choral hydrate. The rat brains were removed and coronally sectioned from −4.3 to +1.7 mm bregma. The right ischemic cortex was separated into its penumbra and ischemic core fractions, and the right penumbral cortex was weighed and homogenized in the cytosolic extraction buffer (#K256-100 BioVision, USA). The lysate was centrifuged at 700×g for 10 min at 4°C, and the supernatant was transferred to a new tube and centrifuged at 10,000×g for 30 min at 4°C. The resulting supernatant was collected and retained as the cytosolic fraction, whereas the pellet was resuspended in 100 µl of the mitochondrial extraction buffer (#K256-100 BioVision, USA) and retained as the mitochondrial fraction. The protein concentrations of the cytosolic and mitochondrial fractions were quantified using a Bio-Rad assay. The samples were boiled at 100°C in a sodium dodecyl sulfate (SDS) gel loading buffer for 10 min prior to loading and running on a 10% SDS polyacrylamide gel. After electrophoresis, the separated proteins were transferred electrophoretically to a nitrocellulose membrane (Hybond-c Extra, Amersham Biosciences, UK) in transfer buffer. The membrane was incubated in 5% skim milk containing 0.1% Tween 20 for 60 min at RT to block nonspecific binding. They were then incubated with a mouse anti-GFAP (1∶1000 dilution, #3670 Cell Signaling Technology), rabbit anti-phospho-SAPK/JNK (p-JNK (Thr183/Tyr185); 1∶1000 dilution, #9251S Cell Signaling Technology), rabbit anti-phospho-p44/42 mitogen-activated protein kinase (MAPK (p-ERK); 1∶1000 dilution, #9101 Cell Signaling Technology), rabbit anti-phospho-p38 MAP kinase (p-p38 MAP kinase (Thr180/Tyr182); 1∶1000 dilution, #9212 Cell Signaling Technology), rabbit anti-cytochrome c (1∶1000 dilution, #4272 Cell Signaling Technology), rabbit anti-tumor necrosis factor receptor type 1-associated death domain (TRADD) (1∶1000 dilution, #3694 Cell Signaling Technology), rabbit anti-Fas-associated death domain (FADD) (1∶1000 dilution, #341282 Calbiochem), rabbit anti-cleaved caspase-8 (1∶1000 dilution, 3259-100 BioVision), or rabbit anti-cleaved caspase-3 (1∶1000 dilution, #9661S Cell Signaling Technology) antibody overnight at 4°C. The transferred membranes were also probed with antibodies specific for mouse anti-actin (1∶5000 dilution, MAB1501 Chemicon), as an internal control for the cytosolic fraction, and mouse anti-cytochrome c oxidase subunit IV (COX IV; 1∶5000 dilution, AB14744-100 Abcam), as an internal control for the mitochondrial fraction, overnight at 4°C. After washing, the membranes were incubated with an anti-rabbit horseradish peroxidase (HRP)-linked IgG (1∶5000 dilution, Jackson ImmunoResearch), anti-mouse HRP-linked IgG (1∶5000 dilution, Santa Cruz Biotechnology), or HRP-conjugated anti-biotin (1∶5000 dilution, Cell Signaling Technology) antibody in a phosphate-buffered saline (PBS) for 1 h at RT. The proteins bands were visualized using an enhanced chemiluminescence reagent (ECL-plus GE Healthcare) on a luminescence image analyzer (LAS-3000, FujiFilm). Densitometric analysis was performed using Alpha Innotech Analyzer software. The optical density was calculated and the levels of proteins were expressed as the densitometric ratio of the proteins to actin or COX IV.

### Statistical Analysis

Data are expressed as mean ± standard deviation (SD). All variables showed approximately normal distribution and the parametric tests, such as analysis of variance (ANOVA) and independent sample *t*-test, were appropriate. The data from all experimental groups were compared using one-way ANOVA followed by post-hoc analysis using the Scheffe test. The percentage cerebral infarct areas and neurological deficit scores within the Sham-1 d and Model-1 d groups were compared using independent sample *t*-test. A *P* value *<*0.05 was considered statistically significant.

## Results

### Effects of EA at Acupoints on the Cerebral Infarct Area

**Figure 1:**
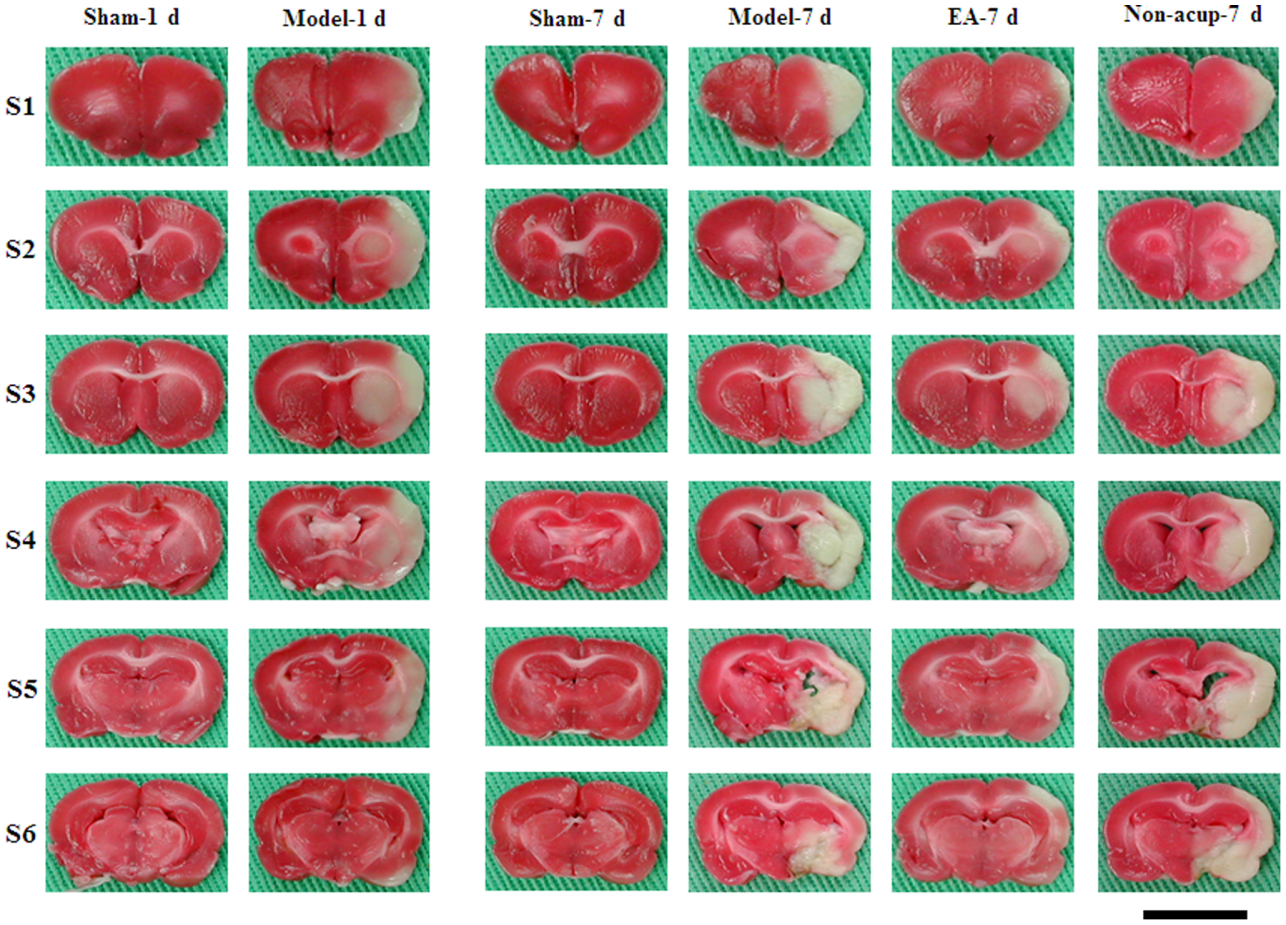
Focal cerebral infarct areas (S1–S6) among the experimental groups following 15 min of ischemia and 1 d or 7 d reperfusion. 2,3,5-Triphenyltetrazolium chloride staining shows the normal brain tissue (red) and infarct tissue (white). Sham-1 d, Sham-1 d group; Model-1 d, Model-1 d group; Sham-7 d, Sham-7 d group; Model-7 d, Model-7 d group; EA-7 d, EA-7 d group; Non-acup-7 d, Non-acup-7 d group. Scale bar = 1 cm.

**Figure 2:**
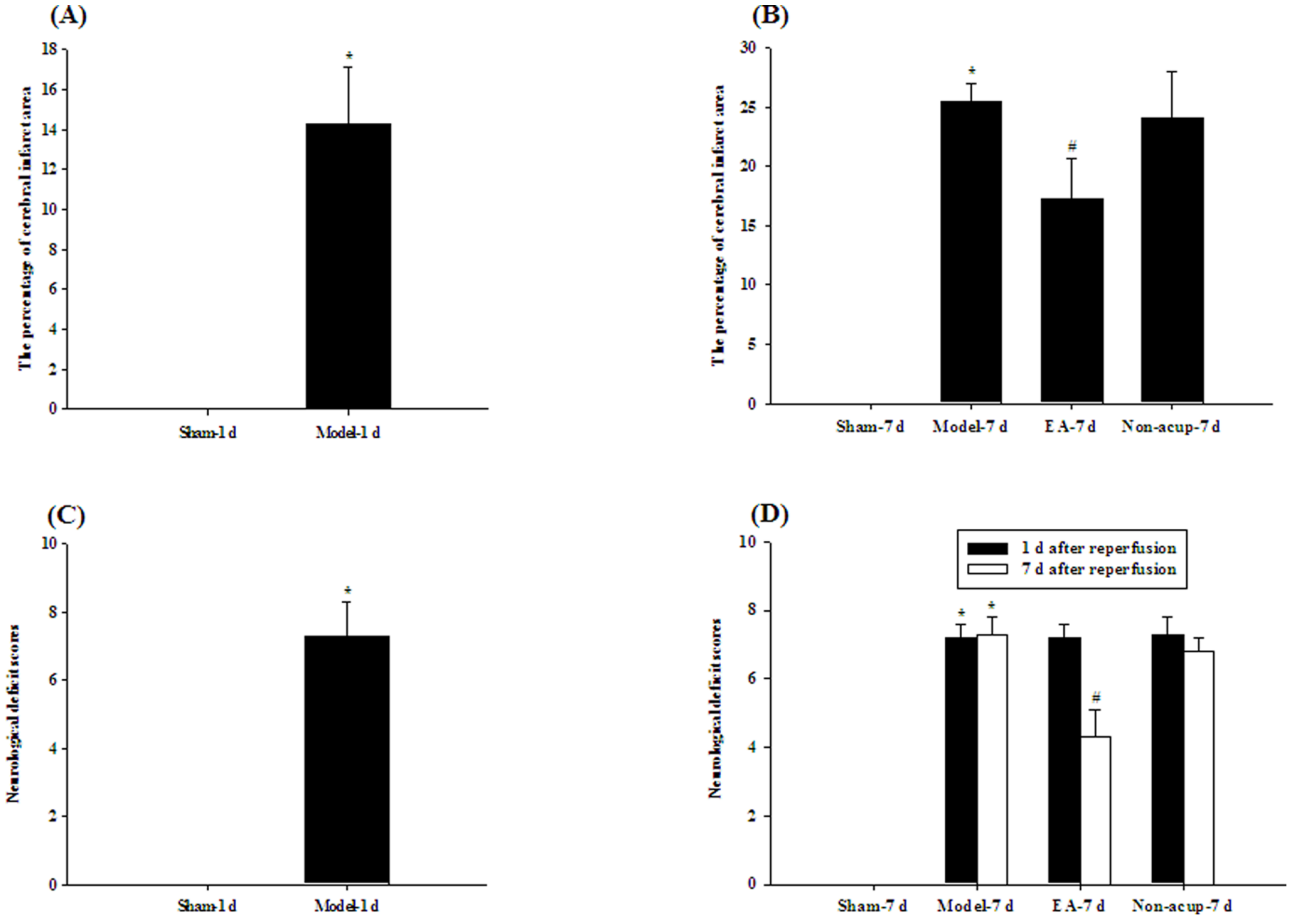
Effects of EA at acupoints on cerebral infarct and neurological status 1 d or 7 d after reperfusion. (A) The percentage cerebral infarct areas in the Sham-1 d and Model-1 d groups were measured at 1 d after reperfusion (n = 5 to 6). (B) The percentage cerebral infarct areas in the Sham-7 d, Model-7 d, EA-7 d, and Non-acup-7 d groups were measured at 7 d after reperfusion (n = 5 to 6). (C) The neurological deficit scores of the Sham-1 d and Model-1 d groups were measured at 1 d after reperfusion. (D) The neurological deficit scores of the Sham-7 d, Model-7 d, EA-7 d, and Non-acup-7 d groups were measured at 1 d and 7 d after reperfusion. Data are presented as mean ± SD. *P<0.05 compared with the Sham-1 d or Sham-7 d group; #*P*<0.05 compared with the Model-7 d group.

The rats showed evidence of cerebral infarct after 15 min of MCAo followed by 1 d of reperfusion (*P*<0.05 vs. Sham-1 d group; Figures 1 and 2A). The percentage cerebral infarct area showed an increasing tendency from 1 d (14.3±2.8%; Model-1 d group) to 7 d (25.5±1.5%; Model-7 d group) after reperfusion (Figures 2A and 2B). Seven days after reperfusion, the percentage cerebral infarct area was significantly higher in the Model-7 d group than in the Sham-7 d group (*P*<0.05), and significantly lower in the EA-7 d group than in the Model-7 d group (*P*<0.05; Figures 1 and 2B). The percentage cerebral infarct areas in the Model-7 d and Non-acup-7 d groups showed non-significant differences (*P*>0.05).

### Effects of EA at Acupoints on Neurological Status

The rats developed moderate neurological deficits after 15 min of MCAo followed by 1 d of reperfusion (7.3±1.0, Model-1 d group; Figure 2C). After 1 d of reperfusion, the neurological deficit scores of the Model-7 d, EA-7 d, and Non-acup-7 d groups showed non-significant differences (*P*>0.05; Figure 2D). After 7 d of reperfusion, the neurological deficit scores were significantly higher in the Model-7 d group than in the Sham-7 d group (*P*<0.05), and significantly lower in the EA-7 d group than in the Model-7 d group (*P*<0.05; Figure 2D). Seven days after reperfusion, the neurological deficit scores of the Model-7 d and Non-acup-7 d groups showed non-significant differences (*P*>0.05; Figure 2D).

### Effects of EA at Acupoints on the Expression of GFAP, S100B, NF-κB (p50), TNF-α, and iNOS

**Figure 3:**
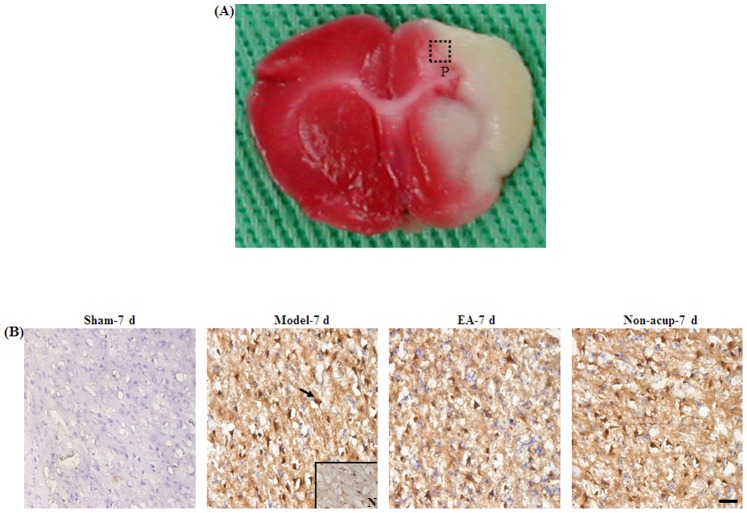
Effects of EA at acupoints on GFAP expression in the ischemic cortical penumbra. (A) Representative photograph shows a TTC-stained brain coronal section 0.92 mm posterior to the bregma. The dotted-line square (1 mm^2^) indicates the area in which the immunoreactive cells were evaluated. (B) Representative photographs show GFAP-immunoreactive cells in the ischemic cortical penumbra in the Sham-7 d, Model-7 d, EA-7 d, and Non-acup-7 d groups 7 d after reperfusion. N, negative control. The arrow indicates a GFAP-immunoreactive cell. Scale bar = 50 µm.

**Figure 4:**
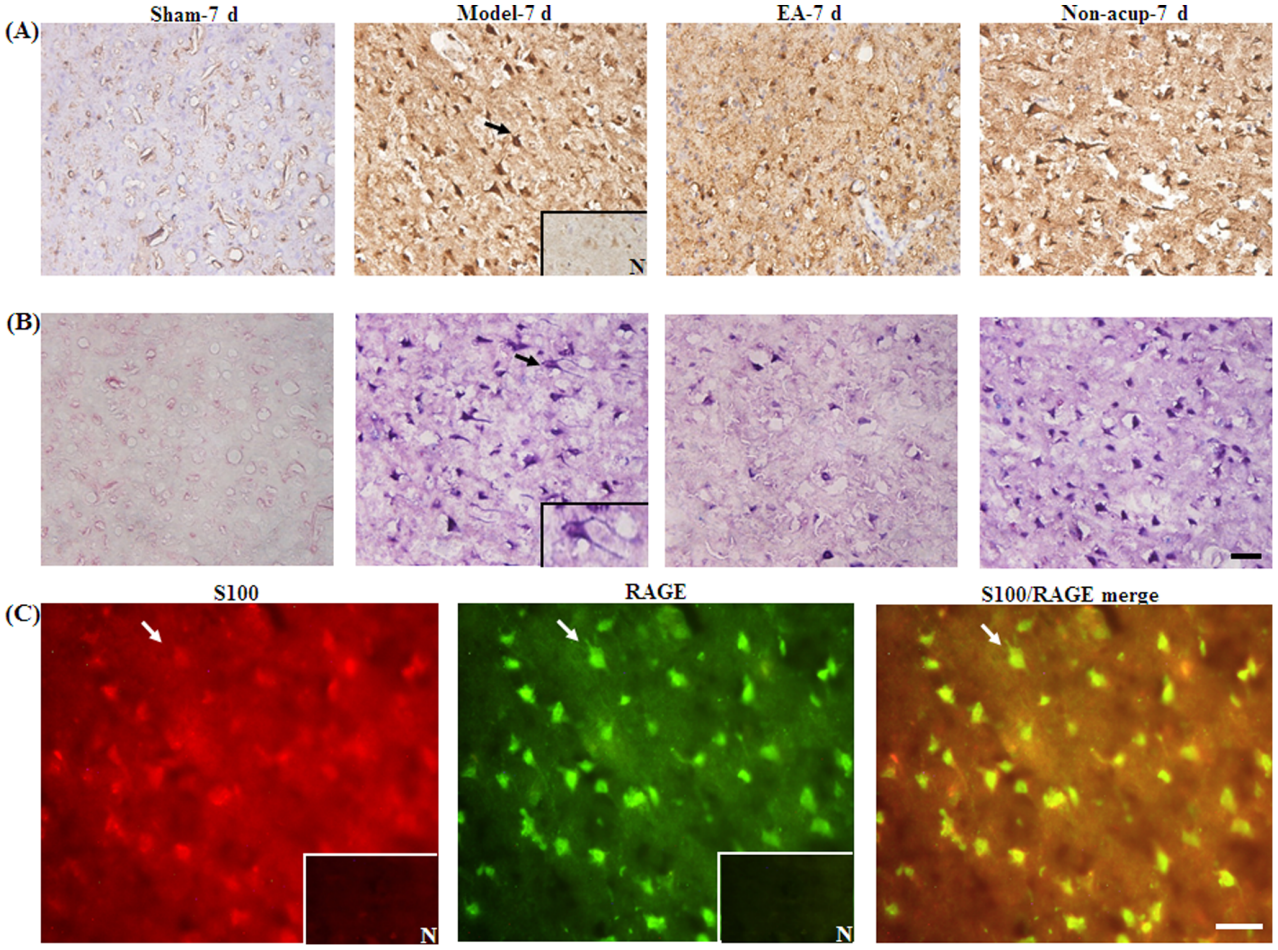
Effects of EA at acupoints on S100B, GFAP/S100B expression, and S100/RAGE expression in the ischemic cortical penumbra. Representative photographs show (A) S100B- and (B) GFAP/S100B-immunoreactive cells in the ischemic cortical penumbra in the Sham-7 d, Model-7 d, EA-7 d, and Non-acup-7 d groups 7 d after reperfusion. (C) Representative photographs show S100-, RAGE-, and S100/RAGE-immunoreactive cells in the ischemic cortical penumbra 7 d after reperfusion. N, negative control. The black arrows in (A) and (B) indicate S100B (brown)- and GFAP/S100B (purple)-immunoreactive cells, respectively. The bottom-right panel shows a GFAP/S100B double-labeled cell at a higher magnification, indicated by a black arrow. The white arrows in (C) indicate S100 (red)-, RAGE (green)-, and S100/RAGE (yellow-green)-immunoreactive cells. Scale bar = 50 µm.

**Figure 5:**
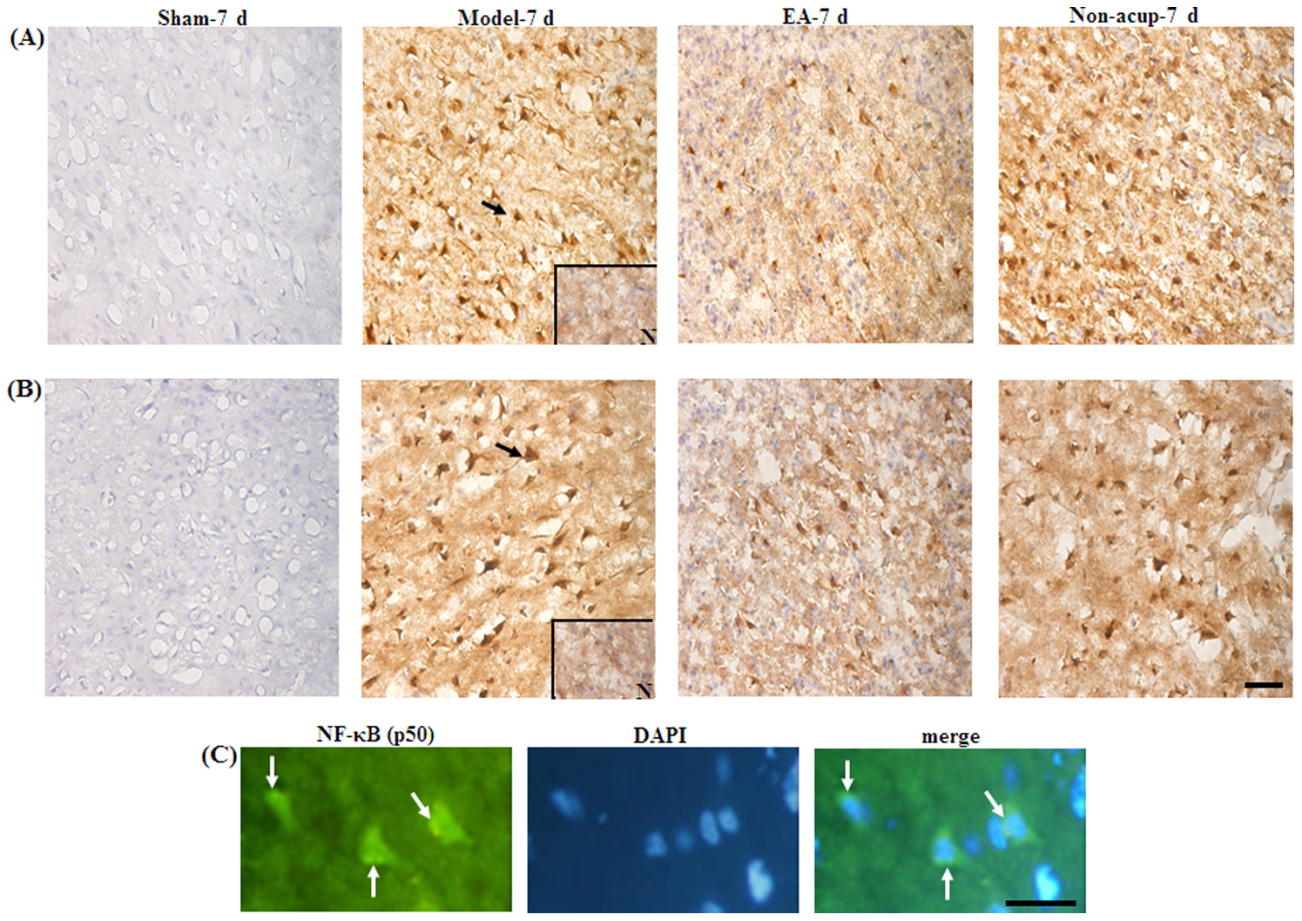
Effects of EA at acupoints on the expression of NF-κB (p50) and TNF-α expression, and the nuclear expression of NF-κB (p50), in the ischemic cortical penumbra. Representative photographs show (A) NF-κB (p50)- and (B) TNF-α-immunoreactive cells in the ischemic cortical penumbra in the Sham-7 d, Model-7 d, EA-7 d, and Non-acup-7 d groups 7 d after reperfusion. (C) Representative photographs show NF-κB (p50)-immunoreactive cells and DAPI-stained nuclei. The merged image shows NF-κB (p50) staining and DAPI-stained nuclei in the ischemic cortical penumbra 7 d after reperfusion. N, negative control. The black arrows in (A) and (B) indicate NF-κB (p50)- and TNF-α-immunoreactive cells, respectively. The white arrows in (C) indicate intense NF-κB (p50) (green) immunoreactivity and NF-κB (p50)/DAPI staining (merged image). Scale bar = 50 µm in (B) and 25 µm in (C).

**Figure 6:**
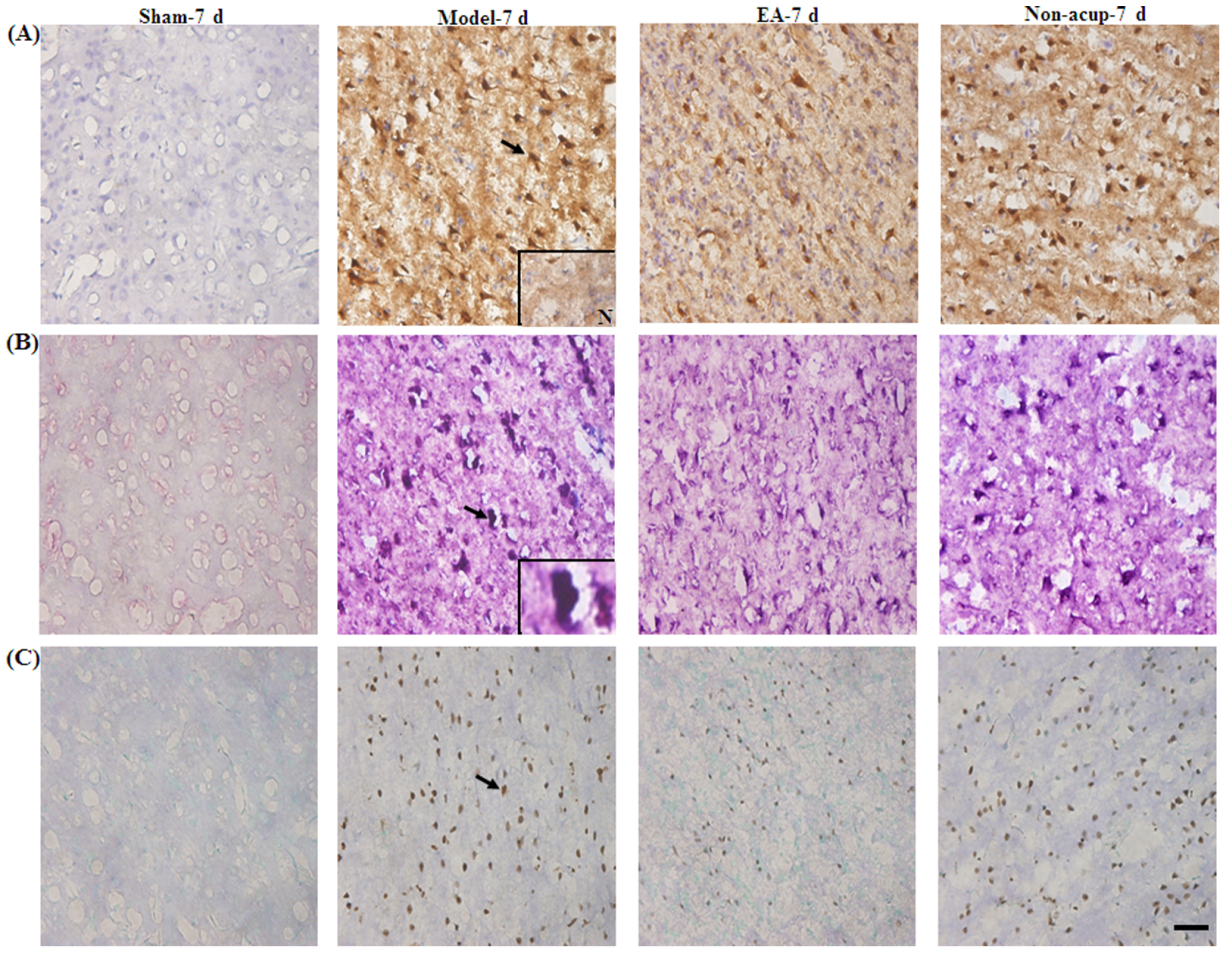
Effects of EA at acupoints on the expression of iNOS, S100B/nitrotyrosine, and TUNEL in the ischemic cortical penumbra. Representative photographs show (A) iNOS-, (B) S100B/nitrotyrosine-, and (C) TUNEL-immunoreactive cells in the ischemic cortical penumbra in the Sham-7 d, Model-7 d, EA-7 d, and Non-acup-7 d groups 7 d after reperfusion. N, negative control. The black arrows in (A), (B), and (C) indicate iNOS (brown)-, S100B/nitrotyrosine (deep purple)-, and TUNEL (brown)-immunoreactive cells, respectively. The bottom-right panel shows a S100B/nitrotyrosine double-labeled cell at a higher magnification, indicated by a black arrow. Scale bar = 50 µm.

We evaluated all GFAP-, S100B-, NF-κB (p50)-, TNF-α-, and iNOS-immunoreactive cells within the dotted line-square in the ischemic cortical penumbra of the brain coronal sections (counts/mm^2^; Figure 3A). After 7 d of reperfusion, the numbers of GFAP-, S100B-, NF-κB (p50)-, TNF-α-, and iNOS-immunoreactive cells were significantly higher in the Model-7 d group than in the Sham-7 d group (all *P*<0.05), and significantly lower in the EA-7 d group than in the Model-7 d group (all *P*<0.05; Figures 3B, 4A, 5A, 5B, and 6A, and Table 1). However, the numbers of GFAP-, S100B-, NF-κB (p50)-, TNF-α-, and iNOS-immunoreactive cells in the Model-7 d and Non-acup-7 d groups showed non-significant differences (all *P*>0.05; Figures 3B, 4A, 5A, 5B, and 6A, and Table 1). Our results from S100/RAGE IF costaining indicated the colocalization of S100 and RAGE, and a marked increase in S100/RAGE immunoreactivity in the ischemic cortical penumbra after MCAo (Figure 4C). Seven days after reperfusion, our NF-κB (p50)/DAPI IF costaining and NF-κB (p50) IHC staining results indicated that NF-κB (p50) was strongly expressed in the nuclei and intense nuclear NF-κB (p50) immunoreactivity was predominantly expressed in the ischemic cortical penumbra. However, 7 d after reperfusion, nuclear NF-κB (p50) immunostaining was considerably less intense in the EA-7 d group than in the Model-7 d group (Figures 5A and 5C).

**Table 1:** The numbers of GFAP-, S100B-, NF-κB (p50)-, TNF-α-, iNOS-, and TUNEL-immunoreactive cells in the ischemic cortical penumbra 7 d after reperfusion (counts/mm^2^).

	Sham-7 d	Model-7 d	EA-7 d	Non-acup-7 d
GFAP	0±0	370±43*	201±43#	428±79
S100B	0±0	440±52*	165±27#	449±30
NF-κB (p50)	0±0	444±31*	200±48#	458±57
TNF-α	0±0	463±39*	182±18#	431±36
iNOS	0±0	433±66*	188±42#	422±59
TUNEL	0±0	172±57*	61±17#	221±43

n = 4–6.

Mean ± SD.

*p<0.05 compared with the Sham-7 d group;

# p<0.05 compared with the Model-7 d group.

### Effects of EA at Acupoints on GFAP/S100B and S100B/nitrotyrosine IHC Costaining

Our IHC costaining results showed that S100B colocalized with GFAP and nitrotyrosine. The GFAP/S100B- and S100B/nitrotyrosine-immunoreactive cells were highly expressed in the ischemic cortical penumbra in the Model-7 d and Non-acup-7 d groups. In contrast, we observed low GFAP/S100B and S100B/nitrotyrosine immunoreactivity in the EA-7 d group (Figures 4B and 6B). We also observed that the distribution patterns for GFAP/S100B and S100B/nitrotyrosine immunoreactivity in the ischemic cortical penumbra were the same as those for S100B immunoreactivity in the experiment groups.

### Effects of EA at Acupoints on the Expression of TUNEL-immunoreactive Cells

We observed that the number of TUNEL-immunoreactive cells in the ischemic cortical penumbra was significantly higher in the Model-7 d group than in the Sham-7 d group (*P*<0.05). The number of TUNEL-immunoreactive cells in the ischemic cortical penumbra was significantly lower in the EA-7 d group than in the Model-7 d group (*P*<0.05; Figure 6C and Table 1). The numbers of TUNEL-immunoreactive cells in the Model-7 d and Non-acup-7 d groups showed non-significant differences (*P*>0.05; Figure 6C and Table 1).

### Effects of EA at Acupoints on the Expression of GFAP, p-JNK, p-ERK, p-p38 MAP Kinase, Cytochrome c, TRADD, FADD, Cleaved Caspase-8, and Cleaved Caspase-3

**Figure 7:**
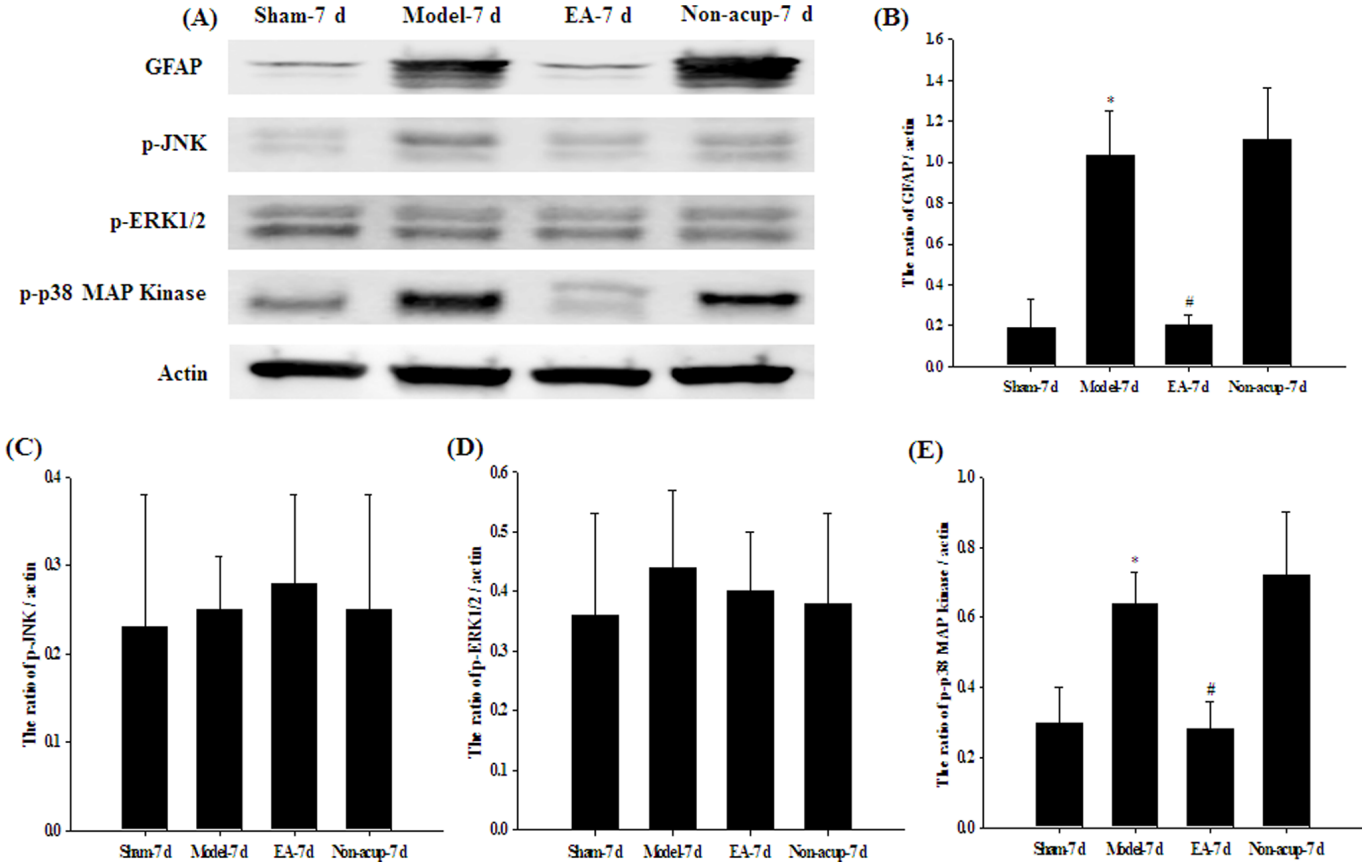
Effects of EA at acupoints on the cytosolic expression of GFAP, p-JNK, p-ERK1/2 and p-p38 MAP kinase in the ischemic cortical penumbra. (A) Representative western blot images show the cytosolic expression of GFAP, p-JNK, p-ERK1/2, and p-p38 MAP kinase in the ischemic cortical penumbra in the Sham-7 d, Model-7 d, EA-7 d, and Non-acup-7 d groups 7 d after reperfusion. Actin was used as an internal control. The relative cytosolic expression of (B) GFAP, (C) p-JNK (D) p-ERK1/2, and (E) p-p38 MAP kinase was evaluated in the ischemic cortical penumbra in the Sham-7 d, Model-7 d, EA-7 d, and Non-acup-7 d groups (n = 4). Data are presented as mean ± SD. *P<0.05 compared with the Sham-7 d group; #*P*<0.05 compared with the Model-7 d group.

**Figure 8:**
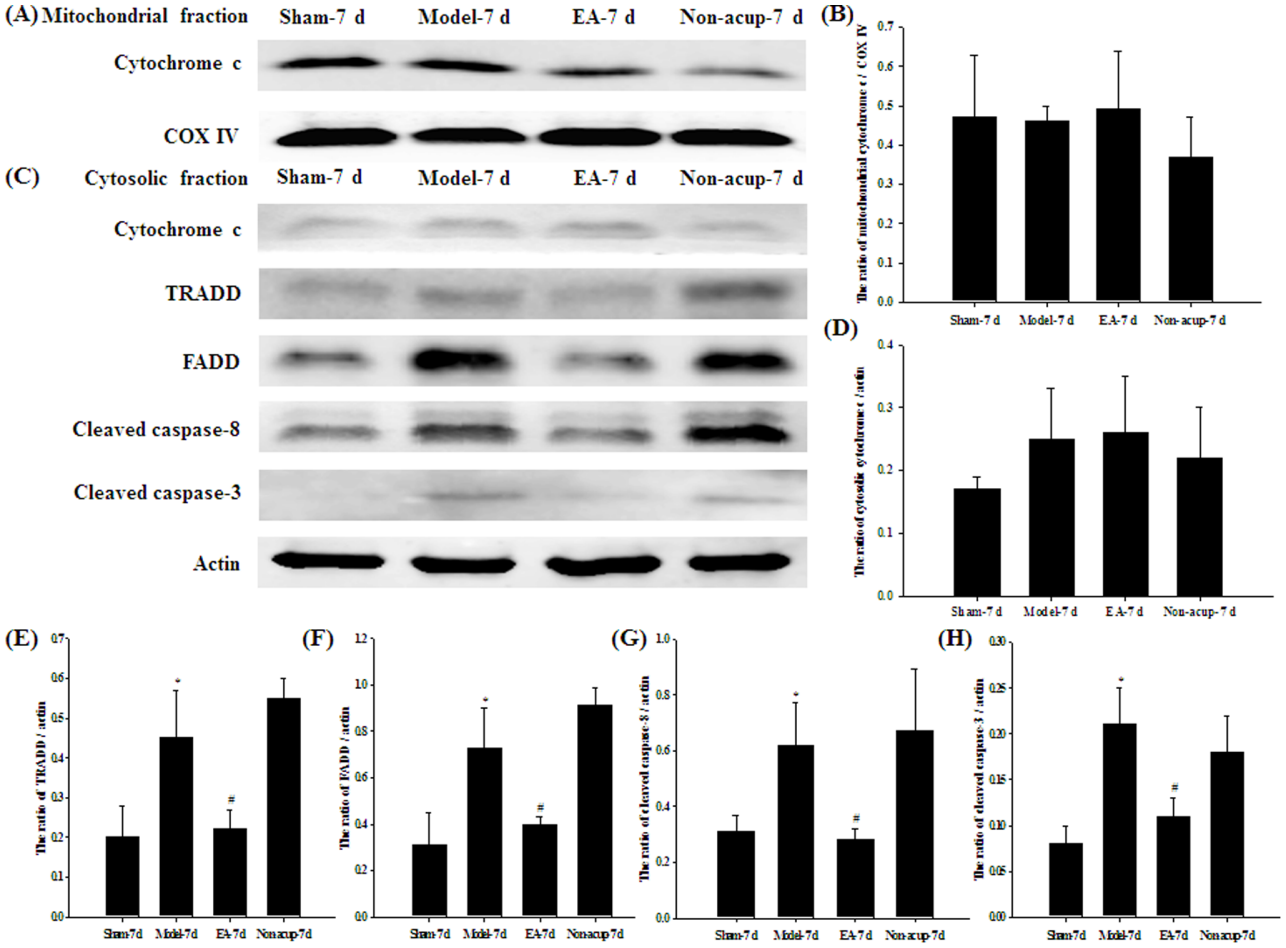
Effects of EA at acupoints on the expression of cytochrome c, TRADD, FADD, cleaved caspase-8, and cleaved caspase-3 in the ischemic cortical penumbra. Representative western blot images show the expression of (A) mitochondrial cytochrome c and (C) cytosolic cytochrome c, TRADD, FADD, cleaved caspase-8 and cleaved caspase-3 in the ischemic cortical penumbra in the Sham-7 d, Model-7 d, EA-7 d, and Non-acup-7 d groups 7 d after reperfusion. COX IV and actin were used as internal controls for the mitochondrial and cytosolic fractions, respectively. The relative mitochondrial expression of (B) cytochrome c, and the relative cytosolic expression of (D) cytochrome c, (E) TRADD, (F) FADD, (G) cleaved caspase-8, and (H) cleaved caspase-3 were evaluated in the ischemic cortical penumbra in the Sham-7 d, Model-7 d, EA-7 d, and Non-acup-7 d groups (n = 4). Data are presented as mean ± SD. *P<0.05 compared with the Sham-7 d group; #*P*<0.05 compared with the Model-7 d group.

Western blot analysis of the ischemic cortical penumbra showed that the cytosolic expression of GFAP and p-p38 MAP kinase was significantly higher (5.4- and 2.1-fold, respectively) in the Model-7 d group than in the Sham-7 d group (*P*<0.05), and significantly lower (0.2- and 0.5-fold, respectively) in the EA-7 d group than in the Model-7 d group (*P*<0.05; Figures 7A, 7B, and 7E). The cytosolic expression of GFAP and p-p38 MAP kinase in the Model-7 d and Non-acup-7 d groups showed non-significant differences (*P*>0.05). In addition, the cytosolic expression of p-JNK and p-ERK in the Sham-7 d, Model-7d, EA-7d, and Non-acup-7 d groups showed non-significant differences (both *P*>0.05; Figures 7A, 7C, and 7D). The mitochondrial (*P*>0.05) and cytosolic (*P*>0.05) expression of cytochrome c in the ischemic cortical penumbra showed non-significant differences among the Sham-7 d, Model-7 d, EA-7 d, and Non-acup-7 d groups (Figures 8A, 8B, 8C, and 8D). The cytosolic expression of TRADD, FADD, cleaved caspase-8, and cleaved caspase-3 in the ischemic cortical penumbra were significantly higher (2.3-, 2.4-, 2.0-, and 2.0-fold, respectively) in the Model-7 d group than in the Sham-7 d group (all *P*<0.05), whereas the cytosolic expression of TRADD, FADD, cleaved caspase-8, and cleaved caspase-3 were significantly lower (all 0.5-fold) in the EA-7 d group than in the Model-7 d group (all *P*<0.05; Figures 8C, 8E, 8F, 8G, and 8H). However, the cytosolic expression of TRADD, FADD, cleaved caspase-8, and cleaved caspase-3 in the Model-7 d and Non-acup-7 d groups showed non-significant differences (all *P*>0.05; Figures 8C, 8E, 8F, 8G, and 8H).

## Discussion

In this study, we observed that 15 min of MCAo consistently caused gross infarction 1 d after reperfusion, and then aggravated delayed infarct expansion in the entire MCA territory, including the cortex and striatum, 7 d after reperfusion. Our findings are in agreement with those of previous studies that used a model of mild transient focal cerebral ischemia to show the initiation of delayed infarction 1 d after reperfusion [22], [23]. Our results indicated that EA at acupoints, applied 1 d after cerebral I/R injury and once daily for 6 consecutive days, significantly reduced the cerebral infarct areas and attenuated the neurological deficits, whereas EA at nonacupoints did not attenuate cerebral ischemic injury and neurological behavioral deficits 7 d after reperfusion. Previous studies have shown that repeated or single EA preconditioning at the Baihui acupoint in rats can elicit ischemic tolerance against cerebral I/R injury [24], [25]. Our findings further indicated that a 24-h therapeutic time window for 6 repeated EA-like stimulations at the Baihui and Dazhui acupoints, but not at nonacupoints, provided effective neuroprotection against subacute ischemic brain injury 7 d after reperfusion in a model of mild focal cerebral ischemia.

Previous studies have used rat models of permanent [6], [26] and transient [27] focal cerebral ischemia to show that the occurrence of delayed infarct expansion is closely related to the activation of astrocytes, which release S100B in the periinfarct area from 1 d after ischemia, with peak release 3–7 d after ischemia. Other studies have shown that the inhibition of astrocytic S100B overexpression in the penumbra can prevent delayed infarct expansion and attenuate neurological deficit during the subacute phase of cerebral I/R injury [5], [28]. In our study, we observed that the GFAP (a marker for activated astrocytes) and S100B proteins were predominantly expressed in the ischemic cortical penumbra. Double IHC staining indicated the colocalization of S100B and GFAP immunoreactivity in astrocytes; however, EA at acupoints significantly downregulated the expression of the GFAP (evaluated by both IHC and western blot analysis) and S100B proteins in the ischemic cortical penumbra 7 d after reperfusion. Therefore, we propose that EA at acupoints exerts its neuroprotective effects against delayed infarct expansion and neurological deficits, at least partly, through the downregulation of S100B expression in the periinfarct area during the subacute phase of cerebral ischemia.

Accumulating evidence has shown that the interaction of a micromolar concentration of S100B and RAGE elicits neurotoxic effects through reactive oxygen species (ROS) and the overproduction of NO [1], [9]. The activation of RAGE by its ligand S100B results in the phosphorylation of the MAPK family members, including ERK1/2, JNK, and p38 MAP kinase, and the activation of the NF-κB pathway, which induces the transcription of iNOS and the proinflammatory cytokines in cell culture models in vitro [29], [30] and in a rodent model of focal cerebral ischemia in vivo [31]. It is well-known that NF-κB, which consists of p50 (50 kDa) and p65/RelA (60 kDa) subunits, can be activated by cerebral ischemic insults to dissociate from the inhibitor protein κB (I-κB), thereby allowing free NF-κB to translocate to the nucleus and initiate the transcription of the proinflammatory genes. Intense nuclear NF-κB p50 or p65 IHC staining provides further evidence of the activation of NF-κB during cerebral ischemia [32]. The S100B/RAGE-induced upregulation of NO and TNF-α synthesis in neurons and glial cells causes oxidative stress and neuronal apoptosis, leading to cerebral infarct expansion, which plays a pivotal pathological role during the subacute phase of cerebral ischemia [6], [33], [34]. In our study, double IF staining showed that the RAGE and S100 proteins (including S100B) colocalized in the ischemic cortical penumbra. Intense nuclear NF-κB (p50) immunostaining indicated the activation of NF-κB. Further IHC analysis confirmed the upregulation of the nuclear expression of NF-κB (p50), and expression of iNOS, and TNF-α, in the ischemic cortical penumbra, and indicated that EA at acupoints effectively downregulated nuclear NF-κB (p50) expression, and consequently iNOS and TNF-α expression, 7 d after reperfusion. Western blot analysis showed that the expression of p-p38 MAP kinase was significantly upregulated in the ischemic cortical penumbra after MCAo, and that EA at acupoints significantly downregulated p-p38 MAP kinase expression, but did not affect p-JNK and p-ERK1/2 expression, in the periinfarct area of the cortex 7 d after reperfusion. The studies by Collino et al. [35], [36] showed that the MAPK signaling cascade is closely associated with the activation of NF-κB, and that the phosphorylation of JNK and p38 MAK kinase contributes to NF-κB activation and the subsequent upregulation of iNOS and TNF-α expression in the hippocampus 1 d after reperfusion. However, the S100B-mediated inflammatory inhibitors exert neuroprotective effects against cerebral I/R injury by reducing oxidative stress and suppressing the inflammatory response after transient cerebral ischemia [35], [36]. Considering our and previous findings, we suggest that S100B/RAGE upregulates iNOS and TNF-α expression through p38 MAP kinase-induced NF-κB activation in the ischemic cortical penumbra during the subacute phase of mild cerebral ischemia. Our results further indicate that EA at acupoints prevents delayed infarct expansion by downregulating the S100B-mediated amplification of the inflammatory response in the periinfarct area of the cortex, and that the effects of EA at acupoints on NF-κB-mediated iNOS and TNF-α expression can be attributed to the inhibition of p38 MAP kinase activity in the ischemic cortical penumbra 7 d after reperfusion.

Increasing evidence has suggested that the neurotoxic effects of iNOS-derived NO can be attributed to its combination with the superoxide anion, leading to the formation of peroxynitrite, a strong oxidative/nitrative molecule that aggravates cerebral I/R injury [37], [38]. Peroxynitrite nitrates tyrosyl residues in proteins to form nitrotyrosine protein adducts, which are biomarkers of peroxynitrite action and exert detrimental effects through the inhibition of tyrosine phosphorylation and impairment of the mitochondrial respiratory chain [39]. Previous studies have shown that nitrotyrosine immunoreactivity increases 24 h after a neonatal focal cerebral ischemic insult, and then gradually reduces in the ischemic cortex 3–7 d after the insult [40], [41]. Nitrotyrosine also induces the opening of the mitochondrial permeability transition pore to promote cytochrome c release and then trigger caspase-3-mediated apoptosis during cerebral ischemia [40], [41]. Our results from double IHC staining showed a marked increase in the colocalization of S100B and nitrotyrosine immunoreactivity in the ischemic cortical penumbra after MCAo, and that EA at acupoints effectively downregulated S100B/nitrotyrosine immunoreactivity 7 d after reperfusion. Our TUNEL assay results showed that the apoptotic cells were predominantly located in the periinfarct area of the cortex, and that EA at acupoints significantly reduced the numbers of apoptotic cells in the ischemic rim 7 d after reperfusion. Upon further analysis, we observed correlation between the patterns of S100B/nitrotyrosine-immunoreactive and TUNEL-immunoreactive cells in the ischemic cortical penumbra. On the basis of these results we reasonably deduce that the neuroprotective effects of EA at acupoints, resulting from S100B/p38 MAP kinase/NF-κB-mediated iNOS and TNF-α modulation, can be attributed to the attenuation of oxidative/nitrative stress and apoptosis in the ischemic cortical penumbra 7 d after reperfusion.

It is well-known that apoptotic neuronal death exacerbates cerebral infarct in the delayed phase of cerebral I/R injury. Previous studies have characterized 2 major caspase-dependent apoptotic pathways: the intrinsic apoptotic pathway, which involves the cytochrome c-initiated caspase cascade, and the extrinsic apoptotic pathway, which is activated by the death receptors and caspase-8 [42]. The extrinsic apoptotic pathway is triggered by the binding of an extracellular death ligand, such as the Fas ligand (FasL) or TNF-α, to a cell surface death receptor. In a pathogenic process of cerebral I/R injury, excess TNF-α binds to the tumor necrosis factor receptor-1 (TNFR1) to elicit the recruitment of TRADD and FADD. These activities induce caspase-8 activation, which triggers the cleavage of caspase-3, leading to apoptosis [43]. In addition, TNF-α induces the rapid activation of NF-κB, which initiates the transcription of the proinflammatory genes, including iNOS and TNF-α, and amplifies TNF-α-mediated apoptosis to increase brain damage during the subacute phase of transient focal cerebral ischemia [43], [44]. According to our western blot analyses, the expression of cytosolic TRADD, FADD, cleaved caspase-8, and cleaved caspase-3 were significantly upregulated in the ischemic cortical penumbra after MCAo. These apoptosis-related proteins, which function in the TNF-α/death receptor signaling pathway, were effectively downregulated by EA at acupoints 7 d after reperfusion. However, EA at acupoints did not affect the mitochondrial or cytosolic expression of cytochrome c in the periinfarct area of the cortex. Therefore, our results strongly suggest that EA at acupoints exerts neuroprotective effects against S100B-mediated apoptosis through the inhibition of the TNF-α/TRADD/FADD/cleaved caspase-8/cleaved caspase-3 signaling pathway in the ischemic cortical penumbra 7 d after reperfusion. The effects of EA at acupoint on TNF-α modulation could further reduce oxidative/nitrative stress and NF-κB-mediated inflammation during the later stages of cerebral I/R injury. The antiapoptotic effects of EA at acupoints did not involve the modulation of the cytochrome c-mediated activation of caspase during the subacute phase of cerebral ischemia. One possible explanation for these observations is that in the presence of cerebral I/R injury, oxidative stress-induced apoptosis (the cytochrome c-related apoptotic pathway) occurs during the early phase of reperfusion, whereas inflammatory-mediated injury can continue to the later phase of reperfusion [35]. However, further research is required to confirm this hypothesis.

In summary, EA at acupoints initiated 1 d postreperfusion effectively downregulates astrocytic S100B expression to provide neuroprotection against delayed infarct expansion through the modulation of p38 MAP kinase-mediated NF-κB expression. The effects of EA at acupoints on NF-κB-induced iNOS and TNF-α regulation contribute to the attenuation of oxidative/nitrative stress and the downregulation of the TNF-α/TRADD/FADD/cleaved caspase-8/cleaved caspase-3 apoptotic pathway in the ischemic cortical penumbra 7 d after reperfusion. Our results indicate that EA at acupoints could potentially provide a therapeutic strategy during the subacute phase of I/R injury in mild transient cerebral ischemia. Further prospective investigations to evaluate the potential clinical application of EA at acupoints are warranted.
